# ChatGPT for Academic Purposes: Survey Among Undergraduate Healthcare Students in Malaysia

**DOI:** 10.7759/cureus.53032

**Published:** 2024-01-27

**Authors:** Renjith George Pallivathukal, Htoo Htoo Kyaw Soe, Preethy Mary Donald, Renu Sarah Samson, Abdul Rashid Hj Ismail

**Affiliations:** 1 Oral Pathology and Oral Biology, Manipal University College Malaysia, Melaka, MYS; 2 Community Medicine, Manipal University College Malaysia, Melaka, MYS; 3 Oral Medicine and Oral Radiology, Manipal University College Malaysia, Melaka, MYS; 4 Orthodontics, Manipal University College Malaysia, Melaka, MYS; 5 Community Dentistry, Manipal University College Malaysia, Melaka, MYS

**Keywords:** kap, undergraduate, medical students, healthcare education, chatgpt, generative artificial intelligence

## Abstract

Background: The impact of generative artificial intelligence-based Chatbots on medical education, particularly in Southeast Asia, is understudied regarding healthcare students' perceptions of its academic utility. Sociodemographic profiles and educational strategies influence prospective healthcare practitioners' attitudes toward AI tools.

Aim and objectives: This study aimed to assess healthcare university students' knowledge, attitude, and practice regarding ChatGPT for academic purposes. It explored chatbot usage frequency, purposes, satisfaction levels, and associations between age, gender, and ChatGPT variables.

Methodology: Four hundred forty-three undergraduate students at a Malaysian tertiary healthcare institute participated, revealing varying awareness levels of ChatGPT's academic utility. Despite concerns about accuracy, ethics, and dependency, participants generally held positive attitudes toward ChatGPT in academics.

Results: Multiple logistic regression highlighted associations between demographics, knowledge, attitude, and academic ChatGPT use. MBBS students were significantly more likely to use ChatGPT for academics than BDS and FIS students. Final-year students exhibited the highest likelihood of academic ChatGPT use. Higher knowledge and positive attitudes correlated with increased academic usage. Most users (45.8%) employed ChatGPT to aid specific assignment sections while completing most work independently. Some did not use it (41.1%), while others heavily relied on it (9.3%). Users also employed it for various purposes, from generating questions to understanding concepts. Thematic analysis of responses showed students’ concerns about data accuracy, plagiarism, ethical issues, and dependency on ChatGPT for academic tasks.

Conclusion: This study aids in creating guidelines for implementing GAI chatbots in healthcare education, emphasizing benefits, and risks, and informing AI developers and educators about ChatGPT's potential in academia.

## Introduction

Artificial intelligence (AI) chatbots have gained considerable popularity as computer programs that simulate human conversation and respond to natural language queries. These chatbots have found widespread application across various industries, including healthcare, retail, and customer service. The COVID-19 pandemic has increased the popularity of E-learning strategies and was later fuelled by the advancements in AI-supported chatbots [1-3]. Among the various generative AI (GAI) tools available, Chat Generative Pre-Trained Transformer (ChatGPT), Bard, Microsoft Bing, Claude, and Scribe have gained prominence for their ability to generate diverse content in education, catering to personalized learning and creativity [4]. ChatGPT, developed by OpenAI, has made a noteworthy impact on education, receiving positive feedback from users for its ability to enhance learning experiences and language proficiency [5]. However, the integration of AI into education also presents certain challenges, including concerns about privacy, accuracy, bias, reliability, and limitations in replicating human creativity [6-8]. Recent studies have explored ChatGPT's potential in clinical practice, scientific enhancement, and public health analysis [9]. Nevertheless, there is a need for further research to understand the factors that influence ChatGPT adoption and implementation challenges in education, particularly regarding usability, accessibility, cultural influences, and socioeconomic backgrounds.

Furthermore, users expressed reservations about the accuracy of ChatGPT's responses and the prospect of bias in its outputs. Questions about the technology's reliability were raised, accompanied by concerns about unforeseen consequences [10-11]. Recent research [12-15] highlighted the potential of AI as a tool to enhance creativity but also underscored the unique and complex nature of human creativity that may be difficult to replicate AI technology fully. Consequently, researchers emphasize the need for further exploration and judicious deliberation to integrate GAI-based large language models (LLMs) into the educational milieu effectively and responsibly.

The scope of ChatGPT and other GAI chatbots in health care practice and education is reaching way beyond mere LLM to the extent that it aids in diagnosing diseases where specialist doctors failed [16]. Though there are studies that tested the accuracy of ChatGPT among the medical and dental educational fraternity, it is noteworthy that the healthcare students' perceptions regarding its utility for academic purposes received limited attention within the realm of research [2,17,18], particularly in the context of Southeast Asia. The diverse sociodemographic profiles and educational strategies in the region may exert significant influence on the attitudes of prospective healthcare practitioners toward the emerging influence of AI-based tools in delivering quality healthcare, both during their training and in subsequent practice. The present study aims to address these gaps by investigating the perceptions and usage of ChatGPT among healthcare students in tertiary healthcare with a focus on exploring their knowledge, attitudes, and practices regarding its academic use. The survey explored the frequency of chatbot usage, the purposes for which they are used, and the satisfaction level with the support provided by chatbots. Additionally, the survey investigated the association between the independent variables such as age, and gender, and dependent variables such as knowledge, attitude, and practice of ChatGPT. The outcomes of this study will provide insights for AI developers and educators, guiding the formulation of guidelines for chatbot utilization in education.

## Materials and methods

Study design and sample size

A cross-sectional survey design was employed to assess the prevalence and patterns of AI-based chatbot usage among university students. The study was conducted among university students, to extend the research to include other national and international institutions in the future. The study population consisted of university students at Manipal University College Malaysia (MUCM). At present, only one institute was selected for piloting because of validating the questionnaire and surveying before extending the study as an international multi-centered collaborative study. The source population was defined as the university students of the MUCM, and universal sampling was followed. Sample size calculation was performed using the formula for a single population proportion, with a precision error of 5%, a confidence level of 95%, and an estimated proportion of 50%. The minimum sample size required was determined to be 400, considering a non-response rate of 20%. The final sample size was set at 500. Non-probability convenience sampling was used, and third-party recruitment was employed to select participants. Inclusion criteria included students of MUCM who were willing to participate and provide informed consent. Non-MUCM students and the general population were excluded from the study. Ethical approval was obtained from the institutional committee before commencing the study (MUCM/Research and Ethics Committee - 005/2023).

A questionnaire (English) was prepared following focus group discussions and underwent item content validation. To evaluate the questionnaire's validity, we performed various calculations including determining the median value for each item, assessing item ambiguity (IA), measuring percentage agreement (PA), calculating the item content validity index (ICVI), determining the content validity ratio (CVR), and calculating the content validity coefficient (VIk) [19,20]. Questions that did not meet the criteria were removed from the questionnaire. The questionnaire was refined based on the ICVI and expert comments. The final validated questionnaire (Appendix 1, Table 9) consisted of three sections: Knowledge (nine questions), Attitude (11 questions), and Practice (eight questions). An online survey was created in Google Forms using the validated questionnaire and the questionnaire was shared with the appropriate participants through email and social media platforms.

In the knowledge domain, a score of one was assigned for answering “yes,” while a score of zero was given for responding “no.” The cumulative knowledge score was then calculated. As for the attitude toward ChatGPT, a rating of 5 was assigned for “strongly agree” regarding positive statements, while a rating of 1 was given for “strongly disagree.” For negative statements, the scoring was reversed, meaning a rating of 1 was assigned for “strongly agree” and a rating of 5 was assigned for “strongly disagree.” Regarding the practice of ChatGPT for academic purposes, we classified agree as “yes” while disagree or not sure were categorized as “no.”

Microsoft Excel was used for data entry and SPSS version 29 (IBM Corp., Armonk, NY) for data analysis [21]. Descriptive statistics such as frequency and percentage were calculated for categorical data and mean, standard deviation, and range were calculated for quantitative data. For univariable analysis, independent t-test, one-way ANOVA, and chi-square test were used to determine the association between demographic variables and knowledge, attitude, and academic practice of ChatGPT. While employing one-way ANOVA, the assumption of homogeneity was checked, and when the assumption was not met, Welch ANOVA was used. For multivariable analysis, multiple linear regression and multiple logistic regression were performed. The significance level was set at 0.05.

## Results

A total of 443 university students from three different courses Bachelor of Medicine and Bachelor of Surgery (MBBS), Bachelor of Dental Surgery (BDS), and Foundation in Science (FIS) participated in this study. 61.2% of the students were 21-25 years and 70.9% were male. Seven participants opted for “Prefer not to say” regarding the gender (1.5%) (Table 1).

**Table 1:** Demographic characteristics of participants (n = 443).

Variable	N (%)
Age (years)	
18 – 20	162 (36.6)
21 – 25	271 (61.2)
25 – 30	10 (2.3)
Gender*	
Male	314 (70.9)
Female	122 (27.5)
Prefer not to say	7(1.6)
Ethnicity	
Malay	34 (7.7)
Chinese	186 (42.0)
Indian	195 (44.0)
Others	28 (6.3)
Programme	
MBBS	215 (48.5)
BDS	161 (36.3)
FIS	67 (15.1)
Year of study	
Year-1	154 (34.8)
Year-2	79 (17.8)
Year-3	74 (16.7)
Year-4	61 (13.8)
Year-5	75 (16.9)

Participants' knowledge about ChatGPT and its potential uses in academics was assessed, detailing its discovery and capabilities like paraphrasing, generating answers, interpreting data, and suggesting diagnoses. The correct responses are marked with an asterisk (Table 2).

**Table 2:** Knowledge about ChatGPT among the participants (n = 443) **Respondents are aware of tools similar to ChatGPT, such as Quillbot, Bard, Openai, Photomath, Perplexity, Bing chat, Copilot and the like.

Variable	N (%)
How you came to know about ChatGPT?	
News	25 (5.7)
Internet	215 (48.8)
Friends/Family	162 (36.7)
Teachers	39 (8.8)
Do you know that ChatGPT can paraphrase?	
Yes*	319 (72.0)
No	124 (28.)
Do you know that ChatGPT can produce answers to your questions in various formats. (Write an assignment, essay or letter, structured research proposal, prepare a questionnaire and the like)?	
Yes*	367 (82.8)
No	76 (17.2)
Do you know that ChatGPT can interpret data in table, perform statistical test, interpret results and write analysis?	
Yes*	252 (56.9)
No	191 (43.1)
Do you know that ChatGPT can suggest diagnosis based on the symptoms or can solve complex mathematical problems?	
Yes*	278 (62.8)
No	165 (37.2)
Do you know that ChatGPT can generate references in the desired format (Vancouver, Harvard, MLA and the like)?	
Yes*	219 (49.4)
No	224 (50.6)
Do you know that ChatGPT can-do site-specific searches (Pubmed, elicit, google scholar and the like) to avoid fake citations?	
Yes*	213 (48.1)
No	230 (51.9)
Do you know any other similar tools like ChatGPT that can be used for academic purposes?**	
Yes*	96 (21.7)
No	346 (78.3)
Do you know any limitations of ChatGPT?	
Yes*	85 (19.2)
No	357 (80.8)

The present study assessed university students' attitudes toward using ChatGPT for academic purposes (n = 443). Statistical analysis reveals diverse perspectives on ChatGPT's reliability, its impact on academic tasks and integrity, as well as opinions on ethical considerations and the future adoption of AI tools (Table 3).

**Table 3:** Participants' attitudes toward using ChatGPT for academic purposes.

Question	N (%)				
	Strongly agree	Agree	Not sure	Disagree	Strongly disagree
I believe that answers/responses from ChatGPT are reliable and accurate.	26 (5.9)	166 (37.5)	199 (44.9)	44 (9.9)	8 (1.8)
I believe that ChatGPT retrieves the most recent data for generating responses.	42 (9.5)	179 (40.4)	189 (42.7)	28 (6.3)	5 (1.1)
I feel ChatGPT can produce better results/responses than I can do in an examination/assignment.	60 (13.5)	158 (35.7)	168 (37.9)	46 (10.4)	11 (2.5)
I feel the use of ChatGPT by students for academic purpose defeats the purpose of education.	44 (9.9)	96 (21.7)	154 (34.8)	116 (26.2)	33 (7.4)
I believe teachers/subject experts cannot detect assignments written by ChatGPT.	14 (3.2)	61 (13.8)	204 (46.0)	119 (26.9)	45 (10.2)
I believe that using ChatGPT has increased the convenience of completing my academic tasks, it has had an adverse effect on my education/learning.	44 (9.9)	152 (24.3)	158 (35.7)	72 (16.3)	17 (3.8)
I feel using ChatGPT for completing written assignments/examinations is malpractice/cheating.	61 (13.9)	133 (30.2)	147 (33.4)	78 (17.7)	21 (47.7)
I feel it is possible to use ChatGPT to support academic activities without violating ethical concerns.	78 (17.6)	179 (40.4)	162 (36.6)	21 (4.7)	3 (0.7)
I feel the institution should prohibit the use of ChatGPT for academic purposes.	24 (5.4)	41 (9.3)	166 (37.5)	139 (31.4)	73 (16.5)
I believe AI tools like ChatGPT will become the new normal in future.	135 (30.5)	217 (49.0)	85 (19.2)	4 (0.9)	2 (0.5)
I will recommend ChatGPT to my friends for academic purposes.	80 (18.1)	178 (40.2)	145 (32.7)	33 (7.4)	7 (1.6)

The overall knowledge and attitude scores ranged from a mean of 4.1 (SD = 2.3) out of a possible 8 and 37.1 (SD = 3.2) out of 55, respectively (Table 4).

**Table 4:** Knowledge and attitude total score regarding ChatGPT among university students (n = 443)

Variable	Mean (SD)	Minimum – Maximum
Knowledge total score (0 – 8)	4.1 (2.3)	0 – 8
Attitude total score (11 – 55)	37.1 (3.2)	30 – 47

Participants' practice of using ChatGPT for academic and non-academic purposes was evaluated. Students' awareness of institutional regulations and ethical considerations, as well as their intention to continue using ChatGPT for academic purposes, are also highlighted (Table 5).

**Table 5:** Practice of using ChatGPT for academic purposes among the participants (n = 443)

Practice	N (%)
How frequently do you use ChatGPT?	
Daily	18 (4.1)
Weekly	60 (13.5)
Monthly	34 (7.7)
Rarely	331 (74.7)
I use or have used ChatGPT for non-academic purposes like personal projects for fun.	
Agree	216 (48.8)
Not sure	68 (15.3)
Disagree	159 (35.9)
I use or have used ChatGPT to help complete my academic activities.	
Agree	162 (36.6)
Not sure	67 (15.1)
Disagree	214 (48.3)
Use of ChatGPT has significantly reduced time and effort for completing academic work/assignment.	
Agree	292 (65.9)
Not sure	136 (30.7)
Disagree	15 (3.4)
My teachers/institute have prohibited the use of ChatGPT for academic purposes.	
Agree	48 (10.8)
Not sure	249 (56.2)
Disagree	146 (33.0)
My teachers/institutes have specified how to use AI tools like ChatGPT ethically or responsibly	
Agree	105 (23.7)
Not sure	180 (40.6)
Disagree	158 (35.7)
I verify the accuracy of the information or answers given by ChatGPT	
Agree	211 (47.6)
Not sure	185 (41.8)
Disagree	47 (10.6)
I will continue using ChatGPT for academic purposes in the future	
Agree	217 (49.0)
Not sure	172 (38.8)
Disagree	54 (12.2)

Univariable analysis was performed to evaluate the association between gender, year of study and knowledge of ChatGPT. There were significant associations between the program of study, year of study and attitudes and academic use of ChatGPT (Table 6).

**Table 6:** Association between demographic variables and knowledge, attitude and academic use of ChatGPT ^a^Independent t-test; ^b^One-way ANOVA; ^c^Welch ANOVA; ^d^Chi-square. P value less than 0.05 is considered significant, ^e^MBBS - Bachelor of Medicine and Bachelor of Surgery, ^f^BDS-Bachelor of Dental Surgery, ^g^FIS-Foundation In Science

Variable	Knowledge		Attitude		Academic use	
	Mean (SD)	P	Mean (SD)	P	(Yes) N (%)	P
Gender						
Male	3.9 (2.2)	<0.001^a^	37.2 (3.2)	0.667^a^	50 (41.0)	0.222^d^
Female	4.9 (2.2)		37.0 (3.3)		108 (34.4)	
Ethnicity						
Malay	3.6 (2.4)	0.320^b^	37.6 (3.9)	0.511^b^	12 (35.3)	0.723^d^
Chinese	4.1 (2.3)		36.9 (3.1)		63 (33.9)	
Indian	4.3 (2.2)		37.2 (3.2)		77 (39.5)	
Others	4.0 (2.1)		36.8 (3.2)		10 (35.7)	
Programme						
MBBS^e^	4.3 (2.2)	0.225^b^	37.6 (3.5)	0.005^c^	94 (43.7)	0.002^d^
BDS^f^	3.9 (2.3)		36.55(2.9)		42 (26.1)	
FIS^g^	4.1 (2.3)		36.4 (3.0)		26 (38.8)	
Year of study						
Year-1	4.0 (2.2)	<0.001^b^	36.9 (3.2)	0.015^b^	55 (35.7)	<0.001^d^
Year-2	4.8 (2.0)		37.5 (3.4)		29 (36.7)	
Year-3	3.6 (2.5)		36.3 (3.0)		12 (16.2)	
Year-4	4.8 (2.2)		38.1 (3.5)		28 (45.9)	
Year-5	3.7 (2.1)		37.1 (3.0)		38 (50.7)	

Multiple logistic regression analysis was performed to assess associations between program, year of study, knowledge, attitude, and academic use of ChatGPT after adjusting other covariates (Table 7).

**Table 7:** Association between demographic variables, knowledge, attitude, and academic use of ChatGPT Nagelkerke R2 = 25.7% Constant = -10.999 Enter method was applied No multicollinearity Hosmer Lemeshow test, P=01.31 Classification table 70.1% correctly classified Area under Receiver Operating Characteristics (ROC) curve was 76.4% B=regression coefficient; OR = Odds ratio; 95% CI = 95% confidence interval; SE = standard error

Variable	Academic use of Chat GPT			
	Adjusted B	SE	Adjusted OR (95% CI)	P
Gender				
Female	Reference			
Male	0.19	0.25	1.21 (0.74 – 1,98)	0.447
Programme				
BDS	Reference			
MBBS	1.41	0.46	3.13 (1.27 – 7.70)	0.013
FIS	0.69	0.28	1.95 (1.12 – 3.40)	0.018
Year of study				
Year-1	Reference			
Year-2	0.38	0.32	1.38 (0.65 – 2.91)	0.402
Year-3	-0.21	-0.21	0.81 (0.33 – 2.00)	0.645
Year-4	0.46	0.46	1.59 (0.75 – 3.38)	0.226
Year-5	1.22	1.22	3.37 (1.59 – 7.16)	0.002
Knowledge	0.13	0.06	1.14 (1.03 – 1.27)	0.014
Attitude	0.04	0.04	1.27 (1.18 – 1.37)	<0.001

The results of the multiple logistic regression analysis in Table 7 show the association between demographic variables, knowledge, attitude, and academic use of ChatGPT among university students. The students attending the MBBS program (OR = 3.13; 95% CI = 1.27 to 7.7; P = 0.013) and FIS program (OR = 1.95; 95% CI = 1.12 to 3.40; P = 0.018); were more likely to use ChatGPT for academic purpose than those attending BDS program. Regarding the year of study, year-5 students were significantly more likely to use ChatGPT for academic purposes than year-1 students (OR = 3.37; 95% CI = 1.59 to 7.16; P = 0.002). Moreover, the students who had higher knowledge (OR = 1.14; 95% CI = 1.03 to 1.27; P = 0.014) and attitude (OR = 1.27; 95% CI = 1.18 to 1.37; P<0.001) scores were significantly more likely to use ChatGPT.

The logistic regression helps identify the predictors that are significantly associated with the academic use of ChatGPT. The adjusted odds ratio (OR) for male students using ChatGPT for academic purposes compared to female students is 1.21 (95% CI: 0.74 - 1.98), but the p-value is 0.447, which is not statistically significant. This indicates that gender does not have a significant association with the academic use of ChatGPT.

MBBS students have a significantly higher likelihood of using ChatGPT for academic purposes compared to BDS students (adjusted OR = 3.13, 95% CI: 1.27 - 7.70, p = 0.013) and FIS students (adjusted OR = 1.95, 95% CI: 1.12 - 3.40, p = 0.018).

Year-5 students show the highest likelihood of using ChatGPT for academic purposes compared to Year-1 students (adjusted OR = 3.37, 95% CI: 1.59 - 7.16, p = 0.002). Other year groups (Year 2, Year 3, and Year 4) do not show statistically significant associations with academic use of ChatGPT. For each unit increase in knowledge about ChatGPT, there is a 14% increase in the likelihood of using it for academic purposes (adjusted OR = 1.14, 95% CI: 1.03 - 1.27, p = 0.014). This suggests that students with higher knowledge about ChatGPT are more likely to use it for academic tasks. Similarly, for each unit increase in attitude towards ChatGPT, there is a 27% increase in the likelihood of using it for academic purposes (adjusted OR = 1.27, 95% CI: 1.18 - 1.37, p < 0.001). This indicates that students with a more positive attitude towards ChatGPT are more likely to use it academically.

The present study conducted a thematic analysis of the responses received from participants regarding the limitations of ChatGPT for academic use. Themes were identified based on the participants' responses and are quoted as such (Table 8).

**Table 8:** Thematic analysis of the responses received from participants regarding the limitations of ChatGPT for academic use.

Limitations and concerns about using ChatGPT for academic purposes
Citing Fake References:
“It sometimes cites fake references (made-up DOIs/links) for the answers to your questions.”
“Yes, it does not give accurate citations, most of the citations are fake.”
Theme: Reliability of Information
Participants express concerns about ChatGPT occasionally providing inaccurate references, impacting the reliability of information it generates. Some citations are even reported as fake.
Recognition of Visual and Audio:
“Can’t really search for images”
“Can’t recognise visual and audio”
“Text and Image analysis of PDF can be more eloquent.”
Theme: Limitation in Sensory Perception, Information Retrieval and Accuracy
It is noted that ChatGPT cannot recognize visual and audio content, indicating its limitations in sensory perception.
Participants discuss ChatGPT's limitations in searching for images and its potential for providing inaccurate information.
Opinion-Based Answers and Explicit/Illegal Information:
“ChatGPT cannot provide opinion-based answers and explicit or illegal information.”
Theme: Ethical and Legal Boundaries
ChatGPT's inability to provide opinion-based or explicit/illegal information is highlighted, reflecting the ethical and legal guidelines governing its responses.
Accuracy and Possible Inaccuracies:
“It is limited to information until September 2021. It also provides inaccurate answers as it uses websites on the internet to provide answers without verifying their legitimacy.”
“May not provide the most accurate answer sometimes and needed to be corrected.”
Theme: Accuracy and Reliability
Concerns are raised about the accuracy of ChatGPT's responses and the possibility of inaccuracies in the information it generates.
ChatGPT is criticized for occasionally providing incorrect information, emphasizing the importance of accuracy and reliability.
Different Generations of ChatGPT:
“Different generation of ChatGPT have different limitations.”
Theme: Evolution of Capabilities
Acknowledgment that different generations of ChatGPT may have varying limitations, highlighting the evolving capabilities of the model.
Encouraging Manipulation or Self-Change:
“It does not provide advice that encourages manipulation or implies that someone should change themselves.”
Theme: Ethical Guidance
ChatGPT is portrayed as not providing advice that encourages manipulation or self-change, reflecting its ethical guidelines.
Plagiarism and Manipulation:
“Easy to paraphrase and not detectable”
Theme: Misuse of Technology
Concerns about plagiarism and manipulation of ChatGPT's responses by users are mentioned, pointing to the potential for misuse.
Design Limitations:
“Can’t design a website in detail.”
“False information, simple math errors, bugs in generated code”
Theme: Design and technological Capability
ChatGPT's inability to design websites in detail is noted, indicating its limitations in design-related tasks.
Concerns are raised about false information, errors, and bugs in generated code, affecting the reliability of ChatGPT's responses.
Dependency of Students:
” Students will depend too much on it”
Theme: Impact on Education
The dialogue suggests that students may rely on ChatGPT, addressing its potential impact on education.
Limited Information and Verification:
“No real time information.”
Theme: Timeliness and credibility
Concerns are expressed about ChatGPT's limitations in providing up-to-date information and its reliance on websites without verification.
There are concerns about ChatGPT's limitations in terms of the year of information it can access, with users noting that it may not provide the most up-to-date data.
Participants express concerns about the timeliness and accuracy of information, including citations, provided by ChatGPT.
General Responses without Proper Command:
“Your instructions have to be specific enough for it to generate the response if not the reply would just be error.”
“They give very generic answers which sometimes is not the best if you want information.”
“Without proper command, the response of ChatGPT will be too general.”
Theme: Instruction Precision
ChatGPT is described as providing general responses in the absence of clear instructions, highlighting the need for precise commands.
The dialogue emphasizes the need for specific and clear instructions to obtain accurate responses from ChatGPT.
Lack of Personal Touch:
“It’s a robot with no emotions.”
“ChatGPT couldn’t understand humour”
Theme: Human Interaction
Participants note that ChatGPT lacks a personal touch in responses, indicating the absence of human-like interaction.
Persuasion and Wrong Answers:
“Information is not 100% correct, can be persuaded to give a wrong answer.”
Theme: Potential Manipulation
Concerns are raised about ChatGPT being persuaded to give incorrect answers, highlighting the potential for manipulation.
Rationalization of Answers:
“It can’t possibly rationalise any answer it gives”
Theme: Rationalization Capability
ChatGPT's inability to rationalize its answers is noted, indicating limitations in providing explanations.
ChatGPT Manipulation by YouTubers:
“A normal cant manipulates CHATGPT easily but at some point, there are many youtubers which find out that you can manipulate ChatGPT to fulfil your orders and it’s so creepy. I think you can search for it.”
Theme: User Exploitation
The dialogue mentions instances where YouTubers have manipulated ChatGPT to fulfil orders, raising concerns about misuse.
Truth vs. Lies:
“As a large language model, it cannot tell truth from lies. This goes for both writing and checking written work. It has been known to provide citations with the wrong authors, authors who do not exist, papers which do not exist but are attributed to real authors, etc.”
Theme: Credibility and Reliability
ChatGPT's inability to discern truth from lies, both in generating and checking content, is emphasized, impacting its credibility.

## Discussion

The present study results give insights into the students' perceptions and concerns regarding the use of ChatGPT for academic purposes. Demographic variables such as gender, program, and year of study show varying degrees of association with knowledge, attitude, and academic use of ChatGPT among university students. Female students tend to have higher knowledge about ChatGPT, and MBBS students have a more positive attitude and higher academic use compared to other programs. Year of study also influences knowledge, attitude, and academic use, with Year-2 and Year-4 students showing higher scores in these aspects. The regression analysis suggests that program, year of study, knowledge, and attitude toward ChatGPT are significant predictors of its academic use among university students. MBBS students, Year-5 students, and those with higher knowledge and a more positive attitude are more likely to use ChatGPT for academic purposes. However, gender does not seem to have a significant influence on the academic use of ChatGPT in this study.

Knowledge

Students who have used ChatGPT for academic purposes have a good understanding of how it works. They know that it is a large language model (LLM) that can generate text, translate languages, write different kinds of creative content, and answer your questions in an informative way. They also know that ChatGPT is not a human and that it can sometimes make mistakes.

The present study data shows that while a significant proportion of university students are aware of ChatGPT's capabilities, there is still a sizable portion of students who lack knowledge about its various functions and limitations. This indicates the need for better dissemination of information and awareness about AI tools like ChatGPT in educational settings.

Responses regarding knowledge about other tools or methods similar to ChatGPT that can be used for academic purposes showed that they are aware of various tools and methods that can be used for academic purposes, similar to ChatGPT. “Quillbot” is the most frequently mentioned tool, followed by “Bard,” “Openai,” “Photomath,” and others. These tools serve different academic functions, such as generating code scripts, assisting in composing essays, providing ideas for work or study, and more. The cumulative percentage suggests that the listed tools collectively cover the majority of valid responses provided by the respondents.

The open-ended question in the questionnaire “What specific academic tasks have you used ChatGPT for?” revealed the diverse ways in which respondents have used ChatGPT for academic purposes. These tasks include assignment and essay writing, paraphrasing, research proposal writing, searching for study materials, email drafting, and exam preparation. Some respondents also mentioned using ChatGPT for internet surfing, suggesting a wide range of applications beyond traditional academic tasks. Additionally, some responses indicate respondents have not used ChatGPT for academic tasks, have never used it, or do not like using it. These non-use responses were included in the “None of the above” category. These responses highlight the versatility of ChatGPT in assisting with various academic tasks, but they also reflect the diversity of user experiences and preferences, including those who have not utilized it for academic purposes.

Attitude

Table 3 shows attitudes about ChatGPT among the participants. Table 4 shows the mean score of knowledge was 4.1 out of a maximum score of 8 which indicates suboptimal knowledge. The mean attitude score was 37.1 out of maximum score 55 which indicates positive attitudes toward ChatGPT. Notably, a substantial portion of students (ranging from 37.5% to 49.0%) express agreement or strong agreement regarding various aspects of ChatGPT's capabilities, such as its accuracy, data retrieval, and potential to produce better results than themselves in examinations or assignments. Conversely, a considerable percentage (ranging from 17.7% to 56.9%) holds dissenting opinions on topics like ChatGPT's impact on education, the detection of ChatGPT-generated assignments by teachers, and its ethical use in academics.

Interestingly, a majority of students (ranging from 58.0% to 79.5%) foresee a future where AI tools like ChatGPT become commonplace, but there are differing views on whether to recommend ChatGPT to friends for academic purposes, with 58.3% in agreement and 34.2% expressing hesitation.

The data from the present study reveal that while students acknowledge the convenience and potential benefits of ChatGPT, there is also a considerable level of uncertainty, skepticism, and ethical concerns surrounding its use for academic purposes. Many students are unsure about the reliability of responses, its impact on education, and its ethical implications. The findings highlight the need for clear guidelines and ethical discussions around the use of AI tools like ChatGPT in the educational setting.

Previous studies indicate that students are optimistic about the potential of ChatGPT to be used in universities [22,23]. They believe that ChatGPT can be used to help them with their studies, such as providing personalized feedback and answering questions. Students are concerned about the potential for ChatGPT to be used for academic dishonesty and they are aware of the limitations of ChatGPT and the need for careful consideration before it is widely adopted. A recent study [24] observed that there is an emerging consensus among students to use the tool, while educators tend to view its use as plagiarism, highlighting the need for policy discussions on integrating AI into educational frameworks. Students are also generally optimistic about the potential of ChatGPT to be used in universities. These observations are in agreement with the findings of Malik et al. [25] and underscore the importance of considering risk perceptions, usefulness, ease of use, attitudes toward technology, and behavioral factors in the successful implementation of ChatGPT in health care education. 

Practice

Table 5 shows 74.7% of the participants used ChatGPT rarely while 7.7% used it monthly, 13.5% used it weekly and 4.1% used it daily. Moreover, 36.6% of the students used ChatGPT for academic purposes, and 65.9% of the students agreed that using ChatGPT has significantly reduced time and effort in completing academic work. 47.6% of them verified the accuracy of the information given by ChatGPT. 49% of the respondents stated they will continue using ChatGPT for academic purposes in the future. Present study results on the Practice of ChatGPT suggest that ChatGPT is not used frequently, with most students utilizing it rarely. While many students have used ChatGPT for non-academic purposes, a considerable portion agrees that it has helped them with academic tasks and reduced time and effort. Students who have used ChatGPT for academic purposes have used it in a variety of ways. Some students have used it to answer questions about the material they are learning. Others have used it to get feedback on their writing. Still, others have used it to generate ideas for projects or assignments. White et al. [26] reported students who used ChatGPT for an English composition course found that it helped generate ideas and get feedback on their writing. However, they also found that it was important to use ChatGPT in conjunction with other resources, such as human tutors and textbooks.

The question to analyze the extent to which respondents depend on ChatGPT to complete academic tasks revealed varied practices. The majority (45.8%) use it to assist with certain sections of an assignment but still complete most of the work independently. Others mentioned not using ChatGPT for assignments at all (41.1%), opting to complete their assignments without its assistance. There are also responses indicating that some users rely on ChatGPT to complete the majority of their assignments (9.3%) and submit them without making revisions. A few respondents use it for specific purposes like generating multiple-choice questions, getting an estimate about a topic, understanding a theory's general concept, or seeking ideas and paraphrasing them into their own words. Lastly, there are a few respondents who have never used ChatGPT for academic assignments or don't use it at all. This illustrates the diverse ways in which respondents incorporate ChatGPT into their academic workflow, ranging from partial assistance to independent completion of assignments, as well as specific use cases for understanding and generating content.

Results of a survey with 1,000 students over the age of 18 found that 89% admitted to using ChatGPT for homework. Additionally, 48% confessed to using it for at-home tests or quizzes, 50% used it for writing essays, and 22% utilized it for paper outlines [27].

Another study [28] conducted with 1,223 undergraduate and graduate students showed that approximately 30% of college students used ChatGPT for schoolwork during the past academic year. Among these users, almost half reported frequent use of the tool, particularly for English (49%) and “hard” sciences like chemistry and biology (41%) assignments. Moreover, 12% of ChatGPT users reported an increase in their GPA from the fall of 2022 to the spring of 2023.

Welding et al. [29] surveyed 1,000 undergraduate and graduate students and found that 43% of college students have used ChatGPT or a similar AI application. Among the users, 50% stated they had used it to help complete assignments or exams. Approximately 51% of college students considered using AI tools on schoolwork as cheating or plagiarism, while 20% disagreed. However, 57% of college students did not intend to continue using AI tools for their schoolwork.

According to the findings reported by Nietzel et al. in Forbes on March 20, 2023, more than half of college students believe that using ChatGPT, an AI-based chatbot, to complete assignments is considered cheating. The survey highlights the perception among students that relying on such AI tools for academic tasks may violate academic integrity and ethical standards. This indicates that while AI tools like ChatGPT may offer convenience and efficiency in completing assignments, there is also a significant concern among students about their impact on the traditional learning process and academic integrity [30].

The findings of the present study reveal that students have employed ChatGPT for diverse academic tasks, encompassing assignment and essay composition, paraphrasing, research proposal drafting, information retrieval for academic purposes, composing emails, and facilitating exam preparation. Notably, some participants also reported employing ChatGPT for general internet browsing, implying a broad spectrum of applications extending beyond traditional academic functions. In contrast, some respondents indicated that they either had not employed ChatGPT for academic tasks, had never utilized it, or simply did not favor its use. These responses were collectively categorized under the “None of the above” classification. These outcomes underscore the adaptability of ChatGPT in offering support for a wide array of academic responsibilities. Simultaneously, they shed light on the assortment of user encounters and preferences, which encompasses individuals who have abstained from using it for academic purposes.

Risks and limitations of using ChatGPT in academics

Present survey results highlighted various limitations of ChatGPT as perceived by the respondents (Table 8). Some of the common limitations mentioned include inaccuracies in information, issues with citations, the inability to provide real-time data, and the need for specific instructions for generating accurate responses. Users identify these limitations across various aspects of the AI's capabilities, underscoring the importance of understanding its boundaries and using it responsibly in academic and other contexts. Recent studies have brought to light the potential for students to exploit ChatGPT for academic dishonesty. The primary concern lies in the potential compromise to academic integrity presented by AI essay-writing systems, particularly ChatGPT. There is an apprehension that students might submit essays that do not authentically represent their work, thus contravening ethical standards within educational institutions [4,6]. The primary concerns stem from the perceived lack of transparency in OpenAI's technology, which has been deemed an “opaque system.” Additionally, ChatGPT's proficiency in generating synthetic information has raised concerns about its potential to compromise academic integrity [14]. Ethical considerations arise from ChatGPT's ability to closely emulate human-authored content, raising concerns about users mistakenly perceiving interactions with the tool as human-like and thereby introducing ethical complexities. It is emphasized that ChatGPT should not be regarded as a substitute for human expertise due to its ongoing development and susceptibility to errors. Educators are advised to stay abreast of the latest developments in ChatGPT and ensure the implementation of human oversight to facilitate responsible usage in educational contexts [15].

Future directions and recommendations for using GAI in higher education

Present study results indicate despite the uncertainty about its ethical use, teacher/institute guidelines, and the accuracy of responses, a substantial number of students express their intention to continue using ChatGPT for academic purposes in the future. The authors propose the following guidelines based on the present study results for the implementation of GAI chatbot technology in the higher education sector of healthcare:

Develop Comprehensive Awareness Programs

Create programs to enhance students' awareness of AI chatbot technologies, such as ChatGPT, and educate them about the capabilities, limitations, and ethical considerations surrounding the use of such tools in academic settings. This can involve seminars, workshops, or courses dedicated to AI literacy.

Curate Specific Guidelines and Ethical Frameworks

Establish clear guidelines for the ethical use of ChatGPT in academia, emphasizing the importance of academic integrity and discouraging any misuse or reliance that might lead to plagiarism or compromise ethical standards.

Enhance Knowledge and Understanding

Implement strategies to improve students' knowledge of ChatGPT functionalities and limitations, such as integrating AI literacy modules into existing curricula or developing specialized courses addressing AI technologies in education.

Encourage Critical Thinking and Evaluation

Foster critical thinking skills among students to critically assess the information generated by AI chatbots and encourage them to verify and cross-reference the information obtained from ChatGPT with credible sources to ensure accuracy and reliability.

Promote Responsible Use

Encourage responsible and judicious use of ChatGPT as a supplementary tool rather than as a primary source for academic tasks, emphasizing the importance of using it as a complementary resource while maintaining traditional research methods.

Establish Support Systems and Resources

Provide support systems such as guidance from educators, resources for understanding AI technologies, and mechanisms for reporting misuse or concerns related to ChatGPT usage.

Continuous Monitoring and Modification

It is essential to assess the impact of ChatGPT's utilization on academic performance, student learning, and integrity regularly. Based on the findings, the guidelines should be adapted and refined to align with the evolving landscape of AI technologies in education.

Collaborative Efforts

Encourage collaboration among educators, AI developers, and students to ensure that the implementation of ChatGPT aligns with educational objectives while addressing the concerns and needs of the academic community.

Address Concerns and Limitations

This study acknowledges and addresses the concerns and limitations highlighted by students regarding ChatGPT usage. Develop strategies to mitigate these issues through technological advancements or by integrating supplementary tools to complement ChatGPT's functionalities.

Promote Diverse Applications

Encourage students to explore diverse applications of ChatGPT beyond traditional academic tasks. Foster creativity and innovation by showcasing the potential of AI chatbots in various domains within the healthcare and higher education sectors.

Through the implementation of these guidelines, educational institutions can navigate the integration of GAI chatbot technologies, such as ChatGPT, in a manner that supports learning, encourages ethical practices, and maximizes the benefits while mitigating potential risks and concerns associated with their usage in the higher education sector, particularly in healthcare.

Limitations of the study

The present study was conducted with a limited sample size and in a single institute. Since the participants are students, there could be apprehensions to admit that they are using the tool due to the risk of accusation. Conducting more studies with larger and more diverse sampling is crucial to ensure the effective and safe utilization of ChatGPT and other GAI in educational settings. The authors intend to expand the research in the future with a specifically designed questionnaire and with a larger sample size which encompasses university students from countries with diverse cultural backgrounds.

## Conclusions

The findings demonstrated that a significant proportion of the study participants possessed suboptimal knowledge and favorable viewpoints concerning ChatGPT's concept and practical application. Additionally, the research indicated that students with a more positive attitude toward ChatGPT were more inclined to employ it for academic purposes. The majority of students are familiar with Chat GPT and are knowledgeable about its various applications, which function as supplements to conventional resources and enhance their efficiency and productivity when completing daily university tasks. This study highlights the need for further research to address the potential risks associated with using generative AI chatbots, such as ChatGPT, in healthcare academia. However, educators and students should be mindful of the inherent limitations of ChatGPT and use language model software more prudently.

## Appendices

Appendix 1 shows a questionnaire on the knowledge, attitude, and practice of university students toward the Use of ChatGPT for academic purposes.

**Table 9:** Knowledge, attitude, and practice of university students toward the use of ChatGPT for academic purposes

Student: Demographic profile		
Course		
MBBS		
BDS		
FIS		
Other: please specify		
Year of study		
1		
2		
3		
4		
5		
Age		
15-20		
21-25		
25-30		
Above 30		
Sex		
Male		
Female		
Other		
Ethnicity		
Chinese		
Indian		
Malay		
Other: Please specify		
	Knowledge/Awareness	
	How you came to know about ChatGPT? News Internet Friends/Family Teachers Others: Please specify	
	Do you know that ChatGPT can paraphrase? Yes No	
	Do you know that ChatGPT can produce answers to your questions in various formats. (Write an assignment, essay or letter, structured research proposal, prepare a questionnaire and the like)? Yes No	
	Do you know that ChatGPT can interpret data in table, perform statistical test, interpret results and write analysis? Yes No	
	Do you know that ChatGPT can suggest diagnosis based on the symptoms or can solve complex mathematical problems? Yes No	
	Do you know that ChatGPT can generate references in the desired format (Vancouver, Harvard, MLA and the like)? Yes No	
	Do you know that ChatGPT can-do site-specific searches (Pubmed, elicit, google scholar and the like) to avoid fake citations? Yes No	
	Do you know any other similar tools like ChatGPT that can be used for academic purposes? Yes No If yes, please specify	
	Do you know any limitations of ChatGPT? Yes No If yes, please specify	
	Attitude/Perception	
	I believe that answers/responses from ChatGPT are reliable and accurate. Strongly agree Agree Not sure Disagree Strongly disagree	
	I believe that ChatGPT retrieves the most recent data for generating responses. Strongly agree Agree Not sure Disagree Strongly disagree	
	I feel ChatGPT can produce better results/responses than I can do in an examination/assignment. Strongly agree Agree Not sure Disagree Strongly disagree	
	I feel the use of ChatGPT by students for academic purpose defeats the purpose of education Strongly agree Agree Not sure Disagree Strongly disagree	
	I believe teachers/subject experts cannot detect assignments written by ChatGPT. Strongly agree Agree Not sure Disagree Strongly disagree	
	I believe that using ChatGPT has increased the convenience of completing my academic tasks, it has had an adverse effect on my education/learning. Strongly agree Agree Not sure Disagree Strongly disagree	
	I feel using ChatGPT for completing written assignments/examinations is malpractice/cheating. Strongly agree Agree Not sure Disagree Strongly disagree	
	I feel it is possible to use ChatGPT to support academic activities without violating ethical concerns. Strongly agree Agree Not sure Disagree Strongly disagree	
	I feel the institution should prohibit the use of ChatGPT for academic purposes. Strongly agree Agree Not sure Disagree Strongly disagree	
	I believe AI tools like ChatGPT will become the new normal in future Strongly agree Agree Not sure Disagree Strongly disagree	
	I will recommend ChatGPT to my friends for academic purposes. Strongly agree Agree Not sure Disagree Strongly disagree	
	Practice	
	How frequently do you use ChatGPT? Daily Weekly Monthly Rarely	
	I use or have used ChatGPT for non-academic purposes like personal projects for fun Strongly agree Agree Not sure Disagree Strongly disagree	
	I use or have used ChatGPT to help complete my academic activities Strongly agree Agree Not sure Disagree Strongly disagree	
	Use of ChatGPT has significantly reduced time and effort for completing academic work/assignment. Strongly agree Agree Not sure Disagree Strongly disagree	
	My teachers/institute have prohibited the use of ChatGPT for academic purposes. Strongly agree Agree Not sure Disagree Strongly disagree	
	My teachers/institutes have specified how to use AI tools like ChatGPT ethically or responsibly. Strongly agree Agree Not sure Disagree Strongly disagree	
	I verify the accuracy of the information or answers given by ChatGPT. Strongly agree Agree Not sure Disagree Strongly disagree	
	I will continue using ChatGPT for academic purposes in the future. Strongly agree Agree Not sure Disagree Strongly disagree	
